# No association between breast pain and breast cancer: a prospective cohort study of 10 830 symptomatic women presenting to a breast cancer diagnostic clinic

**DOI:** 10.3399/BJGP.2021.0475

**Published:** 2022-02-22

**Authors:** Rajiv V Dave, Hannah Bromley, Vicky P Taxiarchi, Elizabeth Camacho, Sumohan Chatterjee, Nicola Barnes, Gillian Hutchison, Paul Bishop, William Hamilton, Cliona C Kirwan, Ashu Gandhi

**Affiliations:** Nightingale Breast Cancer Centre, Wythenshawe Hospital, Manchester University NHS Foundation Trust, Manchester.; Nightingale Breast Cancer Centre, Wythenshawe Hospital, Manchester University NHS Foundation Trust, Manchester.; Division of Population Health, Health Services Research, and Primary Care, School of Health Sciences, University of Manchester, Manchester, UK.; Division of Population Health, Health Services Research, and Primary Care, School of Health Sciences, University of Manchester, Manchester, UK.; Nightingale Breast Cancer Centre, Wythenshawe Hospital, Manchester University NHS Foundation Trust, Manchester.; Nightingale Breast Cancer Centre, Wythenshawe Hospital, Manchester University NHS Foundation Trust, Manchester.; Nightingale Breast Cancer Centre, Wythenshawe Hospital, Manchester University NHS Foundation Trust, Manchester.; Clinical Sciences, Wythenshawe Hospital, Manchester University NHS Foundation Trust, Manchester.; St Luke’s Campus, University of Exeter, Exeter.; Nightingale Breast Cancer Centre, Wythenshawe Hospital, Manchester University NHS Foundation Trust, Manchester and Manchester Breast Centre, Division of Cancer Sciences, Faculty of Biology, Medicine and Health, University of Manchester, Oglesby Cancer Research Building, Manchester, UK.; Nightingale Breast Cancer Centre, Wythenshawe Hospital, Manchester University NHS Foundation Trust, Manchester and Manchester Breast Centre, Division of Cancer Sciences, Faculty of Biology, Medicine and Health, University of Manchester, Oglesby Cancer Research Building, Manchester, UK.

**Keywords:** breast pain, breast neoplasm, economic, evaluate, mastalgia

## Abstract

©The Authors

**Background:**

Women with breast pain constitute >20% of breast clinic attendees.

**Aim:**

To investigate breast cancer incidence in women presenting with breast pain and establish the health economics of referring women with breast pain to secondary care.

**Design and setting:**

A prospective cohort study of all consecutive women referred to a breast diagnostic clinic over 12 months.

**Method:**

Women were categorised by presentation into four distinct clinical groups and cancer incidence investigated.

**Results:**

Of 10 830 women, 1972 (18%) were referred with breast pain, 6708 (62%) with lumps, 480 (4%) with nipple symptoms, 1670 (15%) with ‘other’ symptoms. Mammography, performed in 1112 women with breast pain, identified cancer in eight (0.7%). Of the 1972 women with breast pain, breast cancer incidence was 0.4% compared with ∼5% in each of the three other clinical groups. Using ‘breast lump’ as reference, the odds ratio (OR) of women referred with breast pain having breast cancer was 0.05 (95% confidence interval = 0.02 to 0.09, *P*<0.001). Compared with reassurance in primary care, referral was more costly (net cost £262) without additional health benefits (net quality-adjusted life-year [QALY] loss −0.012). The greatest impact on the incremental cost-effectiveness ratio (ICER) was when QALY loss because of referral-associated anxiety was excluded. Primary care reassurance no longer dominated, but the ICER remained greater (£45 528/QALY) than typical UK National Health Service cost-effectiveness thresholds.

**Conclusion:**

This study shows that referring women with breast pain to a breast diagnostic clinic is an inefficient use of limited resources. Alternative management pathways could improve capacity and reduce financial burden.

## INTRODUCTION

Every year over 700 000 women are referred to NHS breast clinics within England^1^ with almost a 100% increase in referrals over the past 10 years.^1^ This surge in referral numbers has not resulted in a similar increase in breast cancer diagnosis; during the same time frame, breast cancer registrations in England have increased by 14%.^2^ The increase in referral numbers to breast clinic reflects multiple causations. One common symptom resulting in referral to secondary care is breast pain. Recent audits^3^^,^^4^ suggest that women referred with breast pain constitute >20% of attendees in breast outpatient services.

National guidance on referral from primary care to secondary care for suspected cancer diagnoses does not feature breast pain alone (that is breast pain without additional symptoms such as breast nodularity, breast lump or complaints related to the nipple–areolar complex) as a symptom of concern.^5^ Nevertheless, the commonest reason for referral of women with symptoms of breast pain to breast cancer diagnostic services is a concern, held by both patient and referring practitioner, that symptoms of breast pain may portend an underlying diagnosis of breast cancer.^3^^,^^6^ This concern is reinforced in secondary care by breast clinicians requesting investigations such as mammography on women presenting with breast pain alone, the justification being ‘to exclude underlying malignancy’,^7^ despite the fact that breast pain or tenderness (in the absence of a palpable mass or other suspicious clinical finding) is rarely a symptom of breast cancer.^8^^,^^9^ Previous reports suggest the breast cancer incidence in patients presenting with breast pain alone to be 0–3%,^7^^,^^10^^–^12 but these reviews were largely retrospective and limited by ‘convenience sampling’.

In this study breast cancer incidence was prospectively assessed in women with breast pain alone as part of a cohort of almost 11 000 consecutive women presenting to a breast cancer diagnostic clinic and also the clinical utility of routine imaging assessment in women being referred with breast pain alone was reviewed. An economic analysis to estimate and compare the costs of outcomes associated with referral (to the breast cancer diagnostic clinic) versus reassurance (by the primary care physician) for women with breast pain alone was conducted.

**Table:** How this fits in

Women with breast pain are often anxious that this symptom may represent an underlying breast malignancy and are consequently referred to secondary care to exclude this diagnosis. This study shows that the incidence of breast cancer in women with breast pain alone (no associated symptoms such as breast lumps or nipple discharge) is 0.4%, a figure similar to that seen in asymptomatic women invited for breast screening. Economic analysis confirms that referral of women with breast pain alone to secondary care diagnostic clinics is associated with increased cost but no additional health benefits. Women with breast pain should be reassured that they are at no greater risk of breast cancer than asymptomatic women.

## METHOD

Real-time prospective electronic patient records of all consecutive women attending a large secondary breast diagnostic clinic between 1 April 2019 and 31 March 2020 was collected and was interrogated for: referral reason, clinical, imaging and pathology findings, and clinical outcome.

All patients were categorised into four distinct groups based on symptoms the presence of which was defined by the patient and/or the referring clinician:

- breast lump: all women with symptoms such as ‘lumpiness’ or ‘lump’ in the breast or axilla, with or without associated pain or nipple symptoms. This group was deemed the reference group for comparative analyses;
- nipple symptoms: all women with nipple discharge, nipple distortion or nipple skin changes, with or without associated pain but no lump;
- breast pain — pain (unilateral or bilateral), reported by the patient or referring practitioner as ‘breast pain’ (presumed arising within the breast), with no other breast symptoms, and no history of breast cancer or breast implant surgery. No distinction was made between cyclical and non-cyclical breast pain;
- ‘other’: encompassing any other symptoms not defined above, including any of the following: breast infection, incidental finding on non-breast imaging (for example thoracic computed tomography scan), patients with a previous breast cancer presenting with breast pain alone, patients with breast implants in situ.

Subgroup analysis included age by category (<40 years, 40–73 years and >73 years) in accordance with national symptomatic and screening imaging guidelines.^13^ Clinical, imaging and histopathological assessment scores were prospectively attributed to each patient according to national criteria^14^ (Supplementary Table S1); clinical score P1–P5 (based on clinical assessment), ultrasound (U1–U5) and/or mammogram (M1–M5) score based on radiological appearance, and biopsy results (B1–B5, based on standard pathological assessment criteria). Patients deemed to have a normal/benign clinical examination were classified as P1 or P2. Similarly, for mammography and ultrasound, M1–M2 and U1–U2, respectively, were deemed normal/benign. A score of three or above in any of the clinical or imaging categories instigated further investigation, usually in the form of biopsies. For those diagnosed with a cancer, linked cancer registry data were available, including tumour size, grade, axillary node status, and hormone receptor status.

To check and correct for errors in electronic documentation, the anonymised records of all patients identified to be in the ‘Breast pain’ category were further interrogated, to ensure that clinician-entered free text within the electronic patient record did not volunteer any other breast symptoms. This confirmed accurate allocation of patients within the study group (women referred with breast pain as the solitary breast symptom). Women referred to the diagnostic breast clinic from the NHS breast screening programme following identification of abnormalities in breast screening mammography were not included in the study cohort. The study was registered with the Manchester University Hospital NHS Trust clinical audit department (reference 9221).

### Patient and public involvement

Previous focus group discussions, by the authors of the current study, with patients and the public identified the need for increased evidence around breast disease and understanding of symptoms of breast cancer,^15^ and therefore informed the need for this study. Patients were not involved in the analysis or writing of this study.

### Statistical analysis

The study is reported in accordance with the STROBE guidelines for observational studies.^16^ Analysis was performed to: a) describe the assessment pathway associated with different referral groups (and at differing ages) to the breast diagnostic clinic; and b) determine the prevalence of breast cancer within these different referral groups.

Age is presented by median value (interquartile range [IQR]), all nominal variables are presented by frequency (percentage). χ^2^ tests and logistic regression models examined associations between continuous and nominal variables with diagnosis of breast cancer, initially for univariable and subsequently for multivariable analysis. Analyses were computed using Stata MP (version 16).

### Economic evaluation

**Figure 1.**
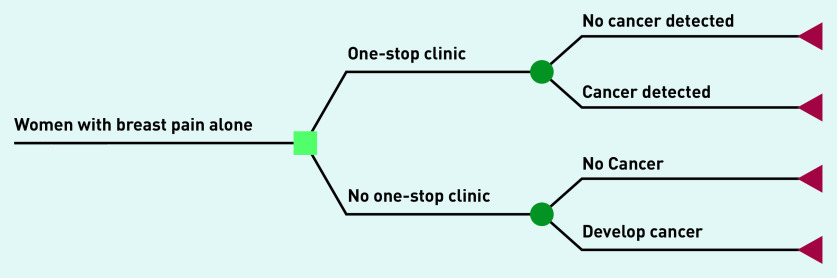
*Model structure to compare costs and outcomes for women with breast pain only referred/not referred to a one-stop breast cancer diagnostic clinic.*

A simple decision model was constructed using a ‘plausible bounds’ approach, similar to previous evaluations of the cost-effectiveness of reforming the UK NHS breast screening programme.^17^ This approach compares a plausible ‘best case’ scenario with a plausible ‘worst-case’ scenario producing a conservative estimate of costs and benefits associated with competing strategies. The decision model was constructed to compare costs and outcomes for women with ‘breast pain only’. The strategies compared were referral to a breast clinic versus reassurance provided by primary care physician with no referral to clinic (Figure 1).

The model was constructed and analysed using TreeAge Pro 2020 software (TreeAge Software, Williamstown, MA). All costs are reported in British pounds (£), price year 2019. An NHS and Social Care perspective was used in the analysis in line with National Institute for Health and Care Excellence (NICE) guidance for economic evaluations of healthcare interventions.^18^^,^^19^ The time horizon for the model was 3 years (156 weeks), reflecting the time between routine screening appointments in the UK NHS breast screening programme for women aged 50–70 years. As the differences in costs and benefits are largely accrued in the first year (that is in relation to the initial clinic visit), no discounting of costs or benefits was included in the model. Data collected at the clinic visit and published literature were used to derive model parameters: costs, utility values and probabilities (presented in Supplementary Table S2). Costs and benefits were combined to report incremental cost-effectiveness ratios (ICERs) in terms of cost per quality-adjusted life-year (QALY) gained for referral versus reassurance. It was necessary to make several assumptions to construct the scenarios in the model; these were explored in one-way sensitivity analyses.

#### Costs

Unit costs were derived from published databases and other published sources including the NHS schedule of reference costs^20^ (summarised in Supplementary Table S2). Costs associated with clinic attendances, investigation, and cancer treatment^21^ were included in the model. Use of healthcare resources (ultrasound scans, mammograms, and biopsies) were derived from breast clinic electronic health records. The model assumed that cancer identified at the diagnostic breast clinic was treated at an early stage, and cancer identified in the no clinic scenario incurred a late-stage treatment cost. An alternative cost, based on breast cancer with a ‘poor’ prognosis, was explored in a sensitivity analysis.^17^

#### Utility/QALYs

As per NICE guidance, the measure of health benefit used in this economic analysis was the QALY.^18^^,^^19^ QALYs combine a measure of health quality (that is utility, measured on a scale from 0 [dead] to 1 [perfect health]), with a measure of time as a single value and were estimated by combining the estimated utility for different levels of health over the duration of the model (156 weeks). The mean age of women in the study cohort was 45 years, therefore a general population utility value for the 45–50-year age group was used in the model.^22^ A utility decrement for anxiety associated with being referred to clinic was included. For women not diagnosed with cancer, the decrement was applied over a 3-week period allowing time for a clinic appointment to occur (typically 2 weeks) and for results of initial diagnostic tests to be available (typically 1 week). For women diagnosed with cancer, the period of anxiety was 4 weeks allowing a longer wait for further diagnostic tests before final diagnosis. It was assumed that the level of the impact on health utility was equivalent to that used in studies of the impact of a false-positive result in routine breast screening programmes. A decrement of 35% for 3 weeks was used in an analysis by Rafia *et al* 23 and as the duration matched the current analysis this level of decrement was used in the primary analysis. Another analysis used a decrement of 5% for 0.2 years (10.4 weeks),^24^ therefore a 5% decrement (over 3 or 4 weeks) was explored in a sensitivity analysis as a lower plausible bound and 50% (over 3 or 4 weeks) was explored as a higher plausible bound. Sensitivity analysis assumed that there was no utility decrement associated with being referred to the clinic.

Based on the clinic data used in the current study and previous reports,^25^ it was assumed that women presenting with pain only and diagnosed with breast cancer had early-stage disease, received treatment and then, after 52 weeks, returned to general population levels of health utility. As a worst-case scenario, for women with cancer not referred to breast clinic, it was assumed that they had equivalent health utility to women with early-stage cancer for 52 weeks, and then late-stage cancer for 52 weeks, after which they died.

#### Probability of cancer

Clinical outcomes from the breast diagnostic clinic were used to establish the probability that women presenting with ‘breast pain only’ had breast cancer.

#### Probabilistic sensitivity analysis

A probabilistic analysis was conducted whereby the costs and utility values were randomly selected 10 000 times from distributions around the values used in the primary (deterministic) model. This generated a 95% confidence interval (CI) around the mean cost and mean QALYs for each scenario. A gamma distribution was used for costs (with α and χ^2^ derived from the unit costs and the standard deviations (SDs) reported in Supplementary Table S2 — where no SD was available an estimated SD of 20% was used). A beta distribution was used for the general population utility value (with α and β derived from the mean and associated SD reported in Supplementary Table S2). A random seed of 383 (generated using random.org) was used.

## RESULTS

**Figure 2.**
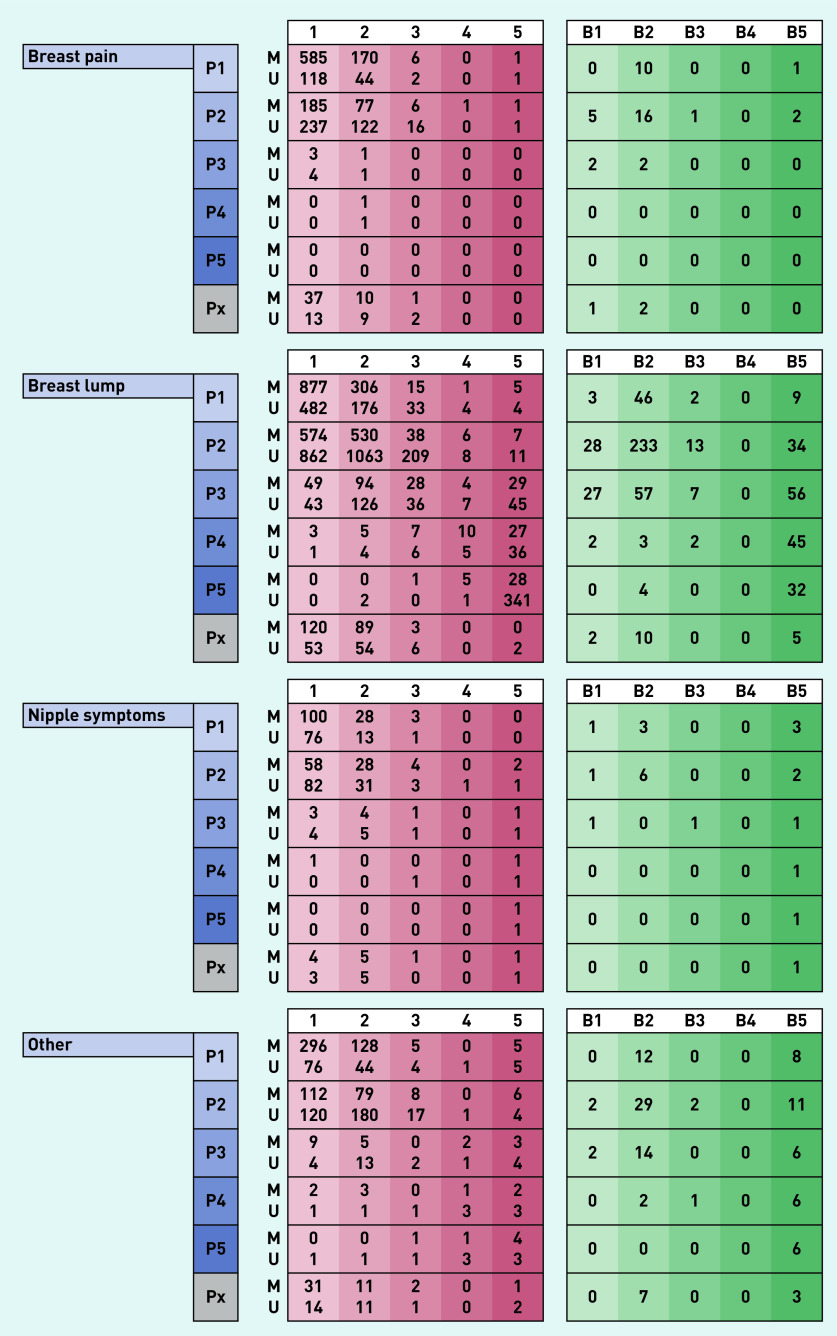
*Clinical and investigation right-side findings in 10 830 women presenting to a new patient breast cancer diagnostic clinic over a 12-month period grouped according to presenting complaint. Scores of 1, 2, 3, 4, and 5 indicate normal, benign, indeterminate, suspicious for malignancy, and malignant, respectively, for each of clinical (P score), mammographic (M score), ultrasound (U score), and histopathological findings (B score). Px = clinical findings not stated.*

**Figure 3.**
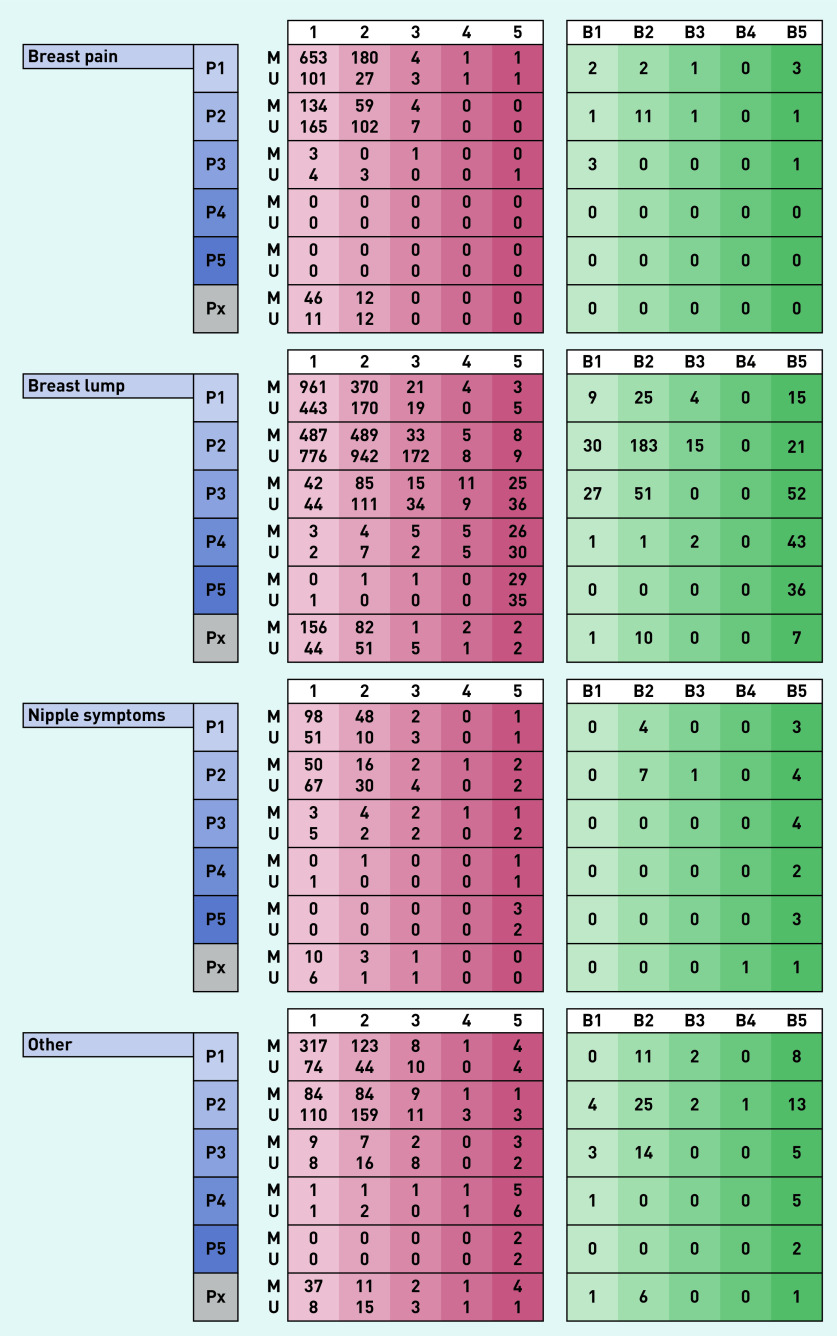
*Clinical and investigation left-sided findings in 10 830 women presenting to a new patient breast cancer diagnostic clinic over a 12-month period grouped according to presenting complaint. Scores of 1, 2, 3, 4, and 5 indicate normal, benign, indeterminate, suspicious for malignancy, and malignant, respectively, for each of clinical (P score), mammographic (M score), ultrasound (U score), and histopathological findings (B score). Px = clinical findings not stated.*

Between 1 April 2019 to 31 March 2020, 10 830 symptomatic women attended the breast clinic. There were 3804 women referred with symptoms that included breast pain (35% of the total cohort), of which 3249 had unilateral pain and 555 had bilateral pain. Patients referred with breast pain alone (*n* = 1972) made up 18% of total referrals to the clinic. The median age of women referred with breast pain was 47 years (IQR 36–60 years), with breast lumps 41 years (IQR 31–52 years), with nipple complaints 45 years (IQR 33–60.5 years) and with other breast symptoms 49 years (IQR 35–63 years) (Supplementary Table S1). Results of the diagnostic assessment and subsequent findings in each group are presented in Figures 2 and 3 and Supplementary Table S1.

### Clinical and radiological findings and incidence of breast cancer

Patients referred with breast pain alone were unlikely to have clinically abnormal findings on breast examination. Just under 1% of women aged <40 years or aged 40–73 years and no one above the age of 73 years had a clinically abnormal (P3–P5) examination finding (Supplementary Table S3). By comparison, in women referred with a breast lump, abnormal (P3–P5) examination findings were present in of 4% in those aged <40 years, 15% aged 40–73 years and 40% aged >73 years (Supplementary Table S3).

Similarly, of the 1112 women with breast pain alone who underwent mammography, this was normal/benign in 98%; mammographically concerning (M3–M5) findings were noted in 2% of women aged 40–73 years and 4% in patients above the age of 73 years (Supplementary Table S3).

For patients referred with breast pain alone, a normal breast examination (P1, P2) had a positive predictive value of 99% (accuracy of 99%) in predicting a normal mammogram (M1, M2), whereas for those presenting with breast lumps, this was 97% (and an accuracy of 92%). For those women referred with breast pain alone proceeding to an ultrasound scan (875 of 1972; 44%), an abnormal finding (U3–U5) was detected in 3% of individuals aged <40 years, 4% aged 40–73 years and 19% aged >73 years (Supplementary Table S3).

The vast majority of women referred with breast pain alone had no indication, based on physical examination to proceed to needle biopsy (1894/1976; 96%; Supplementary Table S1). However, 77 (4% of women presenting with breast pain alone) were found to have incidental radiological findings that led to biopsy with four of these women subjected to multiple biopsies (Supplementary Table S3). Biopsy results were benign in 62 women (81%) and of uncertain malignant potential (B3) in three women. Eight biopsies confirmed breast malignancy (10% of 77 biopsies performed in 1972 women with breast pain alone).

Women referred with a breast lump unsurprisingly had a higher biopsy rate (1217/6708; 18%), of whom 42 (4%) had an indeterminate outcome on biopsy (B3) and 349 (29%) were found to have a breast malignancy. The incidence of pre-invasive or invasive malignancy in women referred with breast pain alone was 0.4%, compared with 5.4% incidence seen in women referred with a breast lump (360/6708), 5.0% with nipple complaints (24/480), and 5.1% with ‘other’ symptoms (86/1670) (Table 1). In the eight women who presented with breast pain alone and were subsequently diagnosed with breast cancer, three had the malignancy diagnosed in the contralateral asymptomatic breast (Supplementary Table S4).

**Table 1.** Incidence of breast cancer within the patient cohort, stratified by referral symptomatology

	**All**	**Breast cancer**	**No breast cancer**	***P*-value**

		**(*n* = 478)**	**(*n*= 10 352)**	
**Age, years, mean (SD)**	45 (16.3)	62 (16.2)	44 (15.9)	<0.001

**Presentation**	***n* (%)**	***n* (%)**	***n* (%)**	<0.001^a^

**Breast pain alone**				
Age, years				
<40		0	647 (100)	
40–73		5 (0.4)	1167 (99.6)	
>73		3 (2.0)	150 (98.0)	
Total^a^	1972 (18.2)	8 (0.4)	1964 (99.6)	

**Breast lump**				
Age, years				
<40		38 (1.2)	3109 (98.8)	
40–73		220 (6.8)	2997 (93.2)	
>73		102 (29.7)	242 (70.3)	
Total^a^	6708 (61.9)	360 (5.4)	6348 (94.6)	

**Nipple complaint**				
Age, years				
<40		1 (0.5)	197 (99.5)	
40–73		14 (6.0)	219 (94.0)	
>73		9 (18.4)	40 (81.6)	
Total^a^	480 (4.4)	24 (5.0)	456 (95.0)	

**Other**				
Age, years				
<40		12 (2.1)	568 (97.9)	
40–73		43 (4.6)	883 (95.4)	
>73		31 (18.9)	133 (81.1)	
Total^a^	1670 (15.4)	86 (5.1)	1584 (94.9)	

a *P-value refers to association between presentation (in total) and breast cancer. SD = standard deviation.*

On multivariable logistic regression modelling women referred with breast pain alone were 20 times less likely to have breast cancer compared with those with breast lumps, after adjustment for age (OR 0.05, 95% CI = 0.02 to 0.09; *P*<0.001) (Table 2). Similarly, women presenting with nipple symptoms (OR 0.59, 95% CI = 0.38 to 0.92) or other breast symptoms (OR 0.56, 95% CI = 0.43 to 0.73) were less likely to have breast cancer compared with women referred with a breast lump. As expected, age was independently associated with breast cancer (OR 1.07 per year of life, 95% CI = 1.07 to 1.08).

**Table 2.** Multivariable logistic regression presenting odds ratio of having a diagnosis of breast cancer based on presentation at the diagnostic clinic, adjusted for age

	**Odds ratio**	**Standard error**	***P*-value**	**95% CI**
**Presentation**				
**Breast lump**	Reference			
**Breast pain alone**	0.05	0.02	<0.001	0.02 to 0.09
**Nipple complaint**	0.59	0.13	0.021	0.38 to 0.92
**Other**	0.56	0.07	<0.001	0.43 to 0.73
**Age**	1.07	0.003	<0.001	1.06 to 1.08

*CI = confidence interval.*

### Economic analysis

The mean cost of a clinic visit in the breast pain group, £269 (95% CI = £265 to £275), was significantly lower than the other presentation groups (lump £361, 95% CI = £356 to £367), nipple complaint £331 (95% CI = £314 to £348), other symptom £322 (95% CI = £312 to £331); *P*<0.05) (Supplementary Table S5).

The total cost of breast clinic attendances for the 1972 women referred with pain alone was £531 817, thus the cost per case of breast cancer identified in this group (*n* = 8) was £66 477. This is around 10-times the cost per case identified in the other presentation groups, which ranged from £6623 to £6944 (Supplementary Table S5).

The results of the base case and sensitivity analyses of the decision model are summarised in Supplementary Table S5. Compared with reassurance in primary care, referral was more costly (net cost £262) and did not confer additional health benefits (net QALYs −0.012) that is referral to secondary care was dominated by reassurance from primary care physicians indicating that it was not likely to be cost-effective (Table 3). The greatest impact on the ICER was when any QALY loss because of anxiety associated with being referred was excluded; primary care reassurance no longer dominated, but the ICER (£45 528/QALY) was still greater than typical cost-effectiveness thresholds used in decision making in the UK NHS. Interventions costing more than £30 000 per QALY gained are not generally considered to be cost-effective.^18^^,^^19^

**Table 3.** Base case and one-way sensitivity analyses for economic evaluation

**Variable**	**Referral group**		**Reassurance group**		**Net cost^a^**	**Net QALYs^a^**	**Cost/QALY**
	
	**Cost, mean (95% CI)**	**QALYs, mean (95% CI)**	**Cost, mean (95% CI)**	**QALYs, mean (95% CI)**			
**Base case**							
Cancer treatment cost if not identified in clinic: £9116							
Anxiety associated with clinic referral: 35% utility decrement for 3 (no cancer) or 4 weeks (cancer)							
QALYs in women with cancer not referred to clinic: 52 weeks early-stage cancer, 52 weeks late-stage cancer, death at 104 weeks Probability of breast cancer with pain only presentation: 0.004							
Deterministic model	£303	2.57	£41	2.59	£262	−0.012	Reassurance dominates
Probabilistic model	£302 (140–538)	2.58 (0–2.98)	£41 (28–57)	2.59 (0–2.99)	£261	−0.011	Reassurance dominates

**Sensitivity analyses on deterministic model**							
Cancer treatment cost – higher (poor prognosis) treatment cost for women not referred to clinic: £15 483	£303	2.57	£67	2.59	£236	−0.012	Reassurance dominates
Anxiety from referral to clinic: none	£303	2.59	£41	2.59	£262	0.006	£45 528
Anxiety from referral to clinic – lower: 5% utility decrement for 3 (no cancer) or 4 weeks (cancer)	£303	2.59	£41	2.59	£262	0.003	£80 430
Anxiety from referral to clinic – higher: 50% utility decrement for 3 (no cancer) or 4 weeks (cancer)	£303	2.57	£41	2.59	£262	−0.019	Reassurance dominates
QALYs in women with cancer not referred to clinic: 52 weeks general population health, 52 weeks late-stage cancer, death at 104 weeks	£303	2.57	£41	2.59	£262	−0.013	Reassurance dominates
QALYs in women with cancer not referred to clinic: 26 weeks early-stage cancer, 26 weeks late-stage cancer, death at 52 weeks	£303	2.57	£41	2.58	£262	−0.010	Reassurance dominates
Probability of breast cancer with pain only presentation – higher in both scenarios: 0.01	£355	2.57	£101	2.58	£254	−0.003	Reassurance dominates

a *As a result of rounding, some net values reported in the table may not be directly calculated from the mean values reported.*

## DISCUSSION

### Summary

This study demonstrated that, in the absence of other breast symptoms, there is no association between breast pain and breast cancer. In this cohort the incidence of breast cancer in women referred with breast pain alone (0.4%) is no higher than the background rate of cancer found in asymptomatic women undergoing breast screening (0.8%).^26^ Referring these women to a breast cancer diagnostic clinic is associated with no demonstrable health benefits but with increased costs and is not an effective use of healthcare resources. The evidence reported here should prompt a review of health policy for the care of women with breast pain alone.

Of women attending the current authors’ breast clinic with breast pain alone, 83% underwent imaging investigations in accordance with national guidance.^13^ Mammography has high negative predictive value in women with breast pain, but positive predictive value for breast cancer is low, 8–14%.^8^ Inevitably, performing imaging investigations in this cohort of women will result in ‘false-positive’ findings; the discovery of benign lesions that would never have caused any symptoms. These are seen in around 5% of women with breast pain alone^27^ and when discovered instigate further intervention. In this cohort, 77 women (4%) experienced the prolonged anxiety of awaiting further investigations and (eventually benign) results, with others reporting similar rates.^27^

In the UK, women aged 50–70 years receive 3-yearly invitations to participate in the NHS breast screening programme where they receive two-view digital mammography. In this cohort of women with breast pain alone 848 (43%) fell into this age category and therefore at the point of referral for breast pain, would likely not be more than a maximum of 18 months from a mammogram (in many cases much closer). Thus, in the absence of any additional breast symptoms, it is improbable that women within the screening age group who develop breast pain alone will benefit from additional mammography outwith routine breast screening.

It is possible that the reassurance provided by attendance at the breast clinic with subsequent normal investigations is in and of itself valuable to women in easing anxiety of an underlying malignant diagnosis. However, data on this is conflicting with arguments both in favour^28^ and against^27^^,^^29^ the ability of normal imaging to provide reassurance to women once a referral to secondary care has occurred. Women, especially younger women, with breast pain undergoing diagnostic imaging with normal results may be more likely to return for further imaging leading to biopsies and health service utilisation than those women receiving reassurance and no imaging at original presentation.^29^ Alternatively, a small study noted that women with breast pain appreciated and were reassured by normal ultrasonography findings.^28^ Further higher-quality research is needed to determine if performing imaging ‘for reassurance’ actually provides the reassurance women seek.

The value of clinician reassurance without imaging for women with breast pain alone has been examined.^30^ Women with breast pain given a verbal explanation for their symptoms and reassurance of the absence of a connection with breast malignancy were evaluated 2–3 months following presentation. In total, 70% of women (85/121) were satisfied with their outcome, reporting lack of progression or resolution of symptoms. A recent systematic review concluded that primary care can be a good location for managing women with breast pain, including assessment of breast cancer risk, provided that healthcare professionals in primary care are supported with well-defined protocols and easy access to secondary care for clinical advice.^10^ Such protocols have been adopted successfully for assessing cardiovascular risk in primary care^31^ and more work is needed to develop and assess similar protocols for breast cancer risk assessment in women with breast pain alone.

### Strengths and limitations

The strengths of this prospective analysis are that it studies an unselected, consecutive cohort of women, investigated in accordance with national guidance.^14^ Contemporaneous recording of outcomes has allowed a largely complete and detailed dataset validated using pre-defined rules and cross-checked with patient records where necessary. Women with benign but treatable causes of breast pain, such as palpable breast cysts, were excluded from the analysis of the breast pain cohort. Women with impalpable breast cysts are unlikely to have pain from such cysts^32^ and are highly unlikely to be a significant presence in the breast pain cohort.

The dataset is limited by the lack of breast cancer family history, which may play a role in directing investigations in a small proportion of women. Furthermore, the data do not include consistent details on other factors relating to breast cancer risk (such as use of oral contraception, hormone replacement therapy, parity, and lactation history). The authors of the current study recognise that cyclical and non-cyclical breast pain may have different aetiology and differing management strategies^33^ but do not make a distinction between the two in this study. However, by capturing all age groups, the current study should capture both clinical scenarios.

This research was an analysis of referral of women with breast pain alone from primary to secondary care. It did not examine the clinical and economic outcomes of managing breast pain from a primary care perspective. Finally, this study was conducted in the referred population and this could lead to spectrum bias,^34^ which is problematic because the procedure of selection for specialist referral produces a different population, usually with higher prevalence of disease than the unselected population. This is less of a concern here, for two reasons. First, as practically all women with breast symptoms in recent years have been referred for exclusion of possible breast cancer, the current referred population closely resembles the unselected population. Second, as the referral process increases the prevalence of the disease of interest, then the likelihood of cancer with breast pain in the unselected population will be lower than in the referred population. These interpretations are supported by the main primary care study of the risk of breast cancer with breast pain, which reported positive predictive values for breast pain of 0.17% (95% CI = 0.16 to 0.17%) in the 40–49-year age group,^9^ compared with the 0.4% in this cohort, with a median age of 47 years.

In the economic evaluation, a plausible bounds approach was used to derive the parameters in the decision tree, similar to a previous economic evaluation of breast cancer screening in the UK NHS.^17^ A benefit of this approach is that it provides an indication of whether more detailed analysis is needed. A limitation is that it aims to produce an estimate of cost-effectiveness and can oversimplify reality. Another area of uncertainty is the negative impact on health utility (in terms of worry or anxiety) that results from being referred to a breast cancer diagnostic clinic as noted in a recent systematic review of the health impact of routine breast screening.^35^ Key assumptions in the model were explored using one-way sensitivity analyses, which had little impact on the ICER. There is little uncertainty that referring women with pain only to a diagnostic breast clinic is not cost-effective, therefore more detailed analysis would lead to little additional knowledge.

### Comparison with existing literature

Previous studies have found cancer detection rates in women referred with breast pain to be 0–3%.^7^^,^^8^^,^^36^ These largely have been smaller retrospective cohorts, unclear on the separate analysis of women with breast pain alone or with associated other breast symptoms. A systematic review of the breast pain literature found the quality of evidence relating to breast pain diagnosis and management to be poor.^10^ Seven of eight studies in the past 10 years note rates of 0–0.4%^8^ with the small numbers of malignancies seen being in women >40 years.^12^^,^^25^^,^^37^ Some suggest that symptoms of non-cyclical focal breast pain may be associated with higher incidence of breast malignancy^25^ than other presentations of breast pain. However, this is inconsistent and has not been noted by others.^36^^–^39 One previous retrospective US study examined health economic costs of evaluating 799 women with breast pain within three breast imaging centres.^27^ It is not clear whether their analysis may be applicable within the UK NHS context.

### Implications for research and practice

Referral of women with breast pain only is not cost-effective and may cause delay for women with higher-risk symptoms. It is an apposite moment to consider more suitable pathways for those women requiring high-quality breast care advice but not a cancer diagnosis service. There is good level II evidence of the value of primary care reassurance and advice as a significant component in the care of women presenting with breast pain alone.^40^ This can be reinforced with online resources.^41^ Most breast pain is self-limiting and will settle in a matter of weeks or months.^6^ In women presenting to primary care with breast pain alone, therefore, deferring referral to secondary care for a period of time may allow spontaneous resolution, averting unnecessary medical intervention for many women without compromising care and enabling more effective use of finite resources.

The findings indicate that women with breast pain alone should be reassured that they have no higher risk of breast cancer than asymptomatic women. They deserve high-quality information and reassurance plus considered advice on how to alleviate their breast pain symptoms. Redirecting women with breast pain alone away from secondary care to more appropriate care pathways will create extra capacity within breast cancer diagnostic clinics for women with true ‘red-flag’ symptoms that have a clear link to breast malignancy.
